# Mechanistic differences in eukaryotic initiation factor requirements for eIF4GI-driven cap-independent translation of structured mRNAs

**DOI:** 10.1016/j.jbc.2024.107866

**Published:** 2024-10-09

**Authors:** Baishakhi Saha, Solomon A. Haizel, Dixie J. Goss

**Affiliations:** 1 Department of Chemistry, Hunter College, City University of New York, New York, New York, USA; 2 PhD. Program in Biochemistry, The Graduate Center of the City University of New York, New York, New York, USA; 3 Center for Genomics and Systems Biology, New York University, New York, New York, USA; 4 PhD. Program in Chemistry, The Graduate Center of the City University of New York, New York, New York, USA

**Keywords:** cap-independent translation initiation, 5′ cap-independent translation enhancer (CITE), internal ribosome entry site (IRES), eIF4GI, eIF4E, eIF4A, structured 5′UTR mRNAs

## Abstract

Protein translation is globally downregulated under stress conditions. Many proteins that are synthesized under stress conditions use a cap-independent translation initiation pathway. A subset of cellular mRNAs that encode for these proteins contain stable secondary structures within their 5′UTR, and initiate cap-independent translation using elements called cap-independent translation enhancers or internal ribosome entry sites within their 5′UTRs. The interaction among initiation factors such as eukaryotic initiation factor 4E (eIF4E), eIF4A, and eIF4GI, especially in regulating the eIF4F complex during noncanonical translation initiation of different 5′UTR mRNAs, is poorly understood. Here, equilibrium-binding assays, CD studies and *in vitro* translation assays were used to elucidate the recruitment of these initiation factors to the highly structured 5′UTRs of fibroblast-growth factor 9 (FGF-9) and hypoxia inducible factor 1 subunit alpha (HIF-1α) encoding mRNAs. We showed that eIF4A and eIF4E enhanced eIF4GI’s binding affinity to the uncapped 5′UTR of HIF-1α mRNA, inducing conformational changes in the protein/RNA complex. In contrast, these factors have no effect on the binding of eIF4GI to the 5′UTR of FGF-9 mRNA. Recently, Izidoro *et al.* reported that the interaction of 42nt unstructured RNA to human eIF4F complex is dominated by eIF4E and ATP-bound state of eIF4A. Here, we show that structured 5′UTR mRNA binding mitigates this requirement. Based on these observations, we describe two possible cap-independent translation mechanisms for FGF-9 and HIF-1α encoding mRNAs used by cells to mitigate cellular stress conditions.

Translation initiation is the rate-limiting step of protein synthesis and is a major target for gene regulation especially during stress conditions (*e.g.*, hypoxia, nutrient deprivation, tumor growth, and so on) (1, 2). Most mRNAs are translated using a cap-dependent pathway. Initial steps in the canonical cap-dependent translation in eukaryotes involve recognition and binding of eukaryotic initiation factor 4E (eIF4E) to the 5′ methyl guanosine (5′ m^7^G) cap of mRNA (3, 4). eIF4E along with eIF4A and the scaffolding protein eIF4GI constitute the eIF4F trimeric complex and together with eIF3, facilitate recruitment of the 43S preinitiation complex (PIC) to the 5′ end of the mRNA to initiate protein synthesis (4, 5, 6). In response to cellular stress conditions, typical cap-dependent translation is compromised as eIF4E is bound and sequestered by the activated 4E binding protein (4E-BP1) (7, 8). However, cells adapt to stress by selectively increasing the expression of a subset of mRNAs while downregulating total protein synthesis using a cap-independent translation pathway (9, 10, 11, 12). The stress-induced translational switch from cap-dependent to cap-independent translation occurs on target mRNAs with structured 5′UTR (13, 14, 15). These mRNAs bypass the requirement for the cap-binding protein, eIF4E, and instead initiate alternate cap-independent translation using structured elements within the 5′UTRs (16, 17). Many cap-independent translations are attributed to internal ribosome entry site (IRES)-like mechanisms, where minimal initiation factors are required, allowing for direct recruitment of ribosomes close to or at the start codon. However, some of the cellular mRNAs require a free 5′end for eIF binding and 43S PIC scanning to initiate translation, known as cap-independent translation enhancer (CITE)-like mechanism (16). Specific secondary structures or sequences within these 5′UTR responsible for this process have not been identified (18). Both these mechanisms are widely researched in viruses (19, 20, 21, 22, 23, 24) but a detailed understanding of similar cap-independent translation mechanisms in cellular mRNAs remains to be discovered.

Further, during these stress conditions, certain protein synthesis factors such as eIF4GI and DAP5 are overexpressed (14, 25, 26). Previous work from our lab demonstrated that a subset of 5′UTR mRNAs, including fibroblast-growth factor 9 (FGF-9) and hypoxia inducible factor 1 subunit alpha (HIF-1α), use IRES-like and CITE-like cap-independent pathway respectively, driven by direct recruitment of eIF4GI to their structured 5′UTR (27). Recent work also reported that the eIF4E binding domain of a truncated eIF4GI_557-1599_ (eIF4GI containing amino acids 557–1599) enhanced its binding affinity to mRNAs by increasing hydrogen bond formation, indicating eIF4GI_557-1599_ as a cap-independent recruitment site for eukaryotic initiation factors (eIFs) by causing conformational rearrangements of complex 5′UTR structures (28). Therefore, the eIF4E binding domain likely serves a dual role in aiding conformational changes of the protein and/or RNA (29) and creating a platform that enhances the ability of eIF4GI to recruit other eIFs (28). The initiation factor requirement for these two possible cap-independent pathways, which have been associated with disease states (*e.g.*, breast cancer, tumor progression, metastasis, diabetic retinopathy, and neurological disorders), remains unknown (15). In this report, we analyzed the effects of additional eukaryotic translation initiation factors eIF4A, eIF4B, and eIF4E on the recruitment of eIF4GI to the 5′UTR of FGF-9 and HIF-1α encoding mRNAs to explore the IRES- and CITE-like translation mechanisms.

eIF4A is an ATP-dependent RNA helicase that binds RNA and ATP cooperatively and unwinds secondary structures within the 5′UTR to facilitate efficient scanning of the 43S PIC to the AUG start codon (30). Generally, the requirement for eIF4A’s helicase activity is dependent on the degree of secondary structure within the 5′UTR of mRNAs (31). eIF4A alone is not efficient in removing these secondary structures, but its unwinding activity is facilitated by eIF4B (32). During cap-independent translation, eIF4A becomes a processive helicase in the presence of eIF4GI and eIF4B (33). It has been recently proposed that eIF4E, known for its cap-binding role (34), may also play a role in the cap-independent translation of highly structured mRNAs by stimulating the unwinding of secondary structures in the 5′UTR (35). Further, eIF4E binding to the autoinhibitory domain of human eIF4GI enhanced the helicase activity of eIF4A (35, 36). For 42nt unstructured RNA, the binding of eIF4E to the eIF4GI•eIF4A stabilized a high-affinity RNA binding state of eIF4F, allowing eIF4A to adopt a dynamic interaction with eIF4GI. This dynamic conformation of eIF4A played an important role in allowing eIF4F to rapidly bind and release mRNA during the scanning process (37). Further, the binding of eIF4GI to eIF4A stabilized a transition between a half open conformation of eIF4A and a fully closed conformation (38). The eIF4A closed conformation was further stabilized by both eIF4GI and eIF4B (39). It has been reported that the presence of eIF4E, nucleotide-bound eIF4A, ATP binding and hydrolysis regulate the binding of eIF4F complex to the 42nt unstructured single-stranded RNA (37). Although the roles of eIF4A, eIF4E, and eIF4B have been extensively studied in the cap-dependent translation initiation mechanism (32, 40), little is known about their roles in cap-independent mechanisms. Therefore, understanding the interactions and functional roles of these initiation factors, along with the role of ATP to facilitate a cap-independent mechanism will increase our understanding of this important alternative protein synthesis mechanism.

In this work, fluorescence-based equilibrium-binding assays, CD studies and *in vitro* translation assays were used to elucidate how initiation factors eIF4GI, eIF4A, eIF4E, and eIF4B are recruited to the uncapped 5′UTRs of FGF-9 and HIF-1α mRNAs, either individually or as a multiprotein complex. We show that eIF4A and eIF4E enhance the binding affinity of eIF4GI to the uncapped 5′UTR of HIF-1α mRNA while having no effect on eIF4GI binding to the uncapped 5′UTR of FGF-9 mRNA. CD spectroscopy indicated distinct structural changes in the eIF4F complex induced by the uncapped 5′UTRs of HIF-1α and FGF-9 mRNAs. Overall, our results identify eIF4A and eIF4E as additional initiation factors necessary for the efficient recruitment of eIF4GI to the uncapped 5′UTR of HIF-1α mRNA, indicating a CITE-like cap-independent translation mechanism. However, these proteins had little to no effect on the cap-independent recruitment of eIF4GI to the uncapped 5′UTR of FGF-9 mRNA. These findings are in contrast with previously reported effects of ATP hydrolysis on a 42-nucleotide unstructured oligonucleotide (37).

## Results

### eIF4A and eIF4E enhance the binding affinity of eIF4GI_557-1599_ to the uncapped 5′UTRs of HIF-1α but not FGF-9 encoding mRNA

**Figure 1:**
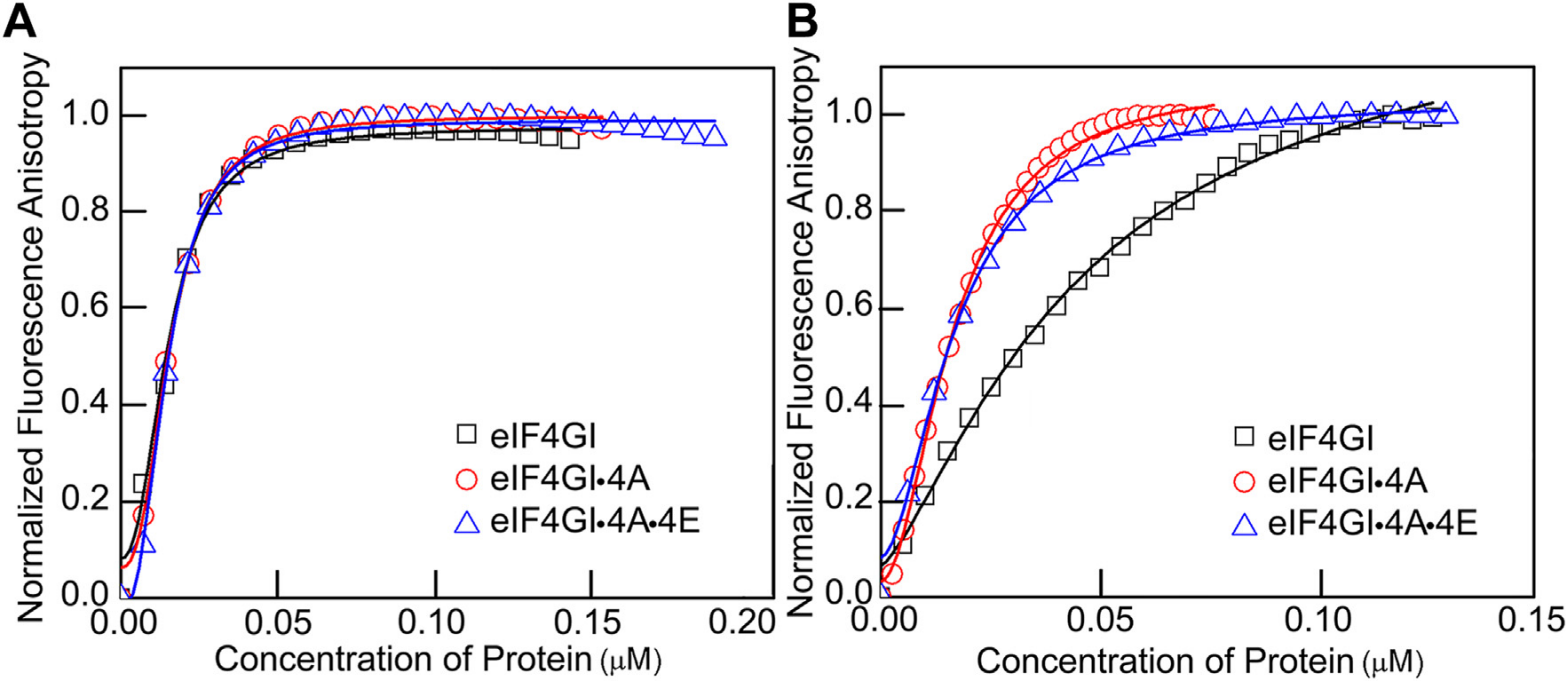
**Comparison of fluorescein-labeled uncapped 5′UTRs of FGF-9 and HIF-1α binding with eIF4G1, eIF4G1-eIF4A, and eIF4G1-eIF4A-eIF4E complexes.** Equilibrium binding titrations of fluorescein-labeled uncapped 5′UTRs of (*A*) FGF-9 and (*B*) HIF-1α mRNAs with eIF4GI_557-1599_ alone, or a mixture of eIF4GI_557-1599_ and eIF4A (eIF4GI_557-1599_•eIF4A) or eIF4GI_557-1599_ and eIF4E (eIF4GI_557-1599_•eIF4E). Briefly, 10 nM fluorescein-labeled uncapped 5′UTR RNA oligonucleotide was titrated with increasing concentrations of protein/protein mixtures in the titration buffer at 25 ± 0.5 °C and the anisotropy at each titration point was measured using excitation and emission wavelengths of 495 nm and 520 nm, respectively. Data points correspond to the average of three independent anisotropy measurements. The curves represent the nonlinear fits used to obtain the corresponding K_D_ values. eIF, eukaryotic initiation factor; FGF, fibroblast-growth factor; HIF-1α, hypoxia inducible factor 1 subunit alpha.

We have previously shown that eIF4E binding domain in eIF4GI_557-1599_ enhances the ability of eIF4GI to recruit other eIFs to the eIF4GI binding site (28). To further investigate the role of other eukaryotic canonical factors eIF4E, eIF4A, and eIF4B in the binding affinities of eIF4GI to the uncapped 5′UTRs of FGF-9 and HIF-1α encoding mRNAs, a fluorescence-based anisotropy assay was performed. This assay was used to determine the binding affinities of eIFs with the uncapped 5′UTRs to understand how they may facilitate mRNA recruitment to eIF4GI for cap-independent translation. Our binding data demonstrate that eIF4A and eIF4E exhibit differential binding affinities to these mRNAs (Table 1). eIF4A bound to uncapped 5′UTRs of FGF-9 and HIF-1α mRNAs with modest affinities of (667 ± 52) nM and (252 ± 20) nM, respectively (Table 1), while eIF4E did not show any appreciable binding to the uncapped 5′UTRs of FGF-9 and HIF-1α mRNAs. To determine how eIF4GI, eIF4A, and eIF4E are recruited to the uncapped 5′UTRs of FGF-9 and HIF-1α mRNAs, either individually or as a multiprotein complex, a series of titrations were performed. The effect of eIF4E and eIF4A in the absence of ATP were assessed by titrating the fluorescein-labeled uncapped RNA oligonucleotides of 5′UTRs of FGF-9 and HIF-1α with eIF4GI_557-1599_ in the presence of eIF4A (final concentration 2.5 μM) (hereafter denoted as eIF4GI_557-1599_•4A) or mixture of eIF4GI_557-1599_ and eIF4E (final concentration 2.5 μM) (hereafter denoted as eIF4GI_557-1599_•4E) or a combination of all three factors, eIF4GI_557-1599,_ eIF4A, and eIF4E (final concentration 2.5 μM) (hereafter denoted as eIF4GI_557-1599_•4A•4E). The dissociation equilibrium constant (K_D_) of eIF4GI_557-1599_•4A for the uncapped 5′UTR of FGF-9 mRNA was calculated to be 16 ± 4 nM as compared to eIF4GI_557-1599_ alone (K_D_, 14 ± 3 nM) (Table 1, Fig. 1*A*), suggesting that eIF4A is not required for the stable recruitment of eIF4GI_557-1599_ to the uncapped 5′UTR of FGF-9 mRNA. For the uncapped 5′UTR of HIF-1α mRNA, the K_D_ value of the eIF4GI_557-1599_•4A complex was 17  ± 2 nM compared to 50  ± 6 nM for eIF4GI_557-1599_ alone (Table 1, Fig. 1*B*), indicating a 3-fold greater affinity for eIF4GI_557-1599_ in the presence of eIF4A compared to eIF4GI_557-1599_ only. Additionally, eIF4GI_557-1599_ binding affinity to uncapped 5′UTR of HIF-1α mRNA was also enhanced by the presence of eIF4E (eIF4GI_557-1599_•4E complex, K_D,_ 15 ± 3 nM) as compared to eIF4GI_557-1599_ alone (K_D,_ 50  ± 3 nM) (Table 1, Fig. 1*B*). This 3-fold increase in binding affinity suggests that eIF4E may induce conformational changes in eIF4GI, facilitating stable recruitment to the uncapped 5′UTR of HIF-1α mRNA. These results are consistent with our previous report where eIF4E induced secondary conformational changes in plant eIF4G, resulting in stronger binding and selectivity to the barley yellow dwarf virus (BYDV) translational element (24). In contrast, the uncapped 5′UTR of FGF-9 mRNA showed no significant improvement in binding affinity with eIF4GI_557-1599_•4E complex, (K_D,_ 16 ± 3 nM) compared to eIF4GI_557-1599_ alone (K_D,_ 14 ± 3 nM) (Table 1, Fig. 1*A*). These observations suggest a crucial role for eIF4E and eIF4A in the cap-independent translation of HIF-1α mRNA. In contrast, the data indicate that eIF4GI_557-1599_ strongly binds to the uncapped 5′UTR of FGF-9 mRNA, eliminating the need for additional eIFs to enhance the binding affinity. Previous studies have shown a correlation between the binding affinity and *in vitro* translation results (27, 28), therefore suggesting the recruitment of eIF4GI_557-1599_ to the uncapped 5′UTR of HIF-1α mRNA, which exhibits weaker binding, likely requires other eIFs for efficient translation initiation.

**Table 1:** Dissociation constant values of uncapped 5′UTR mRNAs binding with eIFs at 25 ± 0.5 °C

Complex bound	FGF-9 (K_D_, nM)	χ^2^	HIF-1α (K_D_, nM)	χ^2^	42 nt CAA^a^ (K_D_, nM)
eIF4GI_557-1599_	14 ± 3	0.991	50 ± 6	0.995	
eIF4A	667 ± 52	0.997	252 ± 20	0.991	
eIF4A + ATP	115 ± 20	0.991	28 ± 5	0.989	
eIF4B	38 ± 8	0.999	54 ± 6	0.998	
eIF4E	NA		NA		
eIF4GI_557-1599_+eIF4E	16 ± 3	0.996	15 ± 3	0.994	ND
eIF4GI_557-1599_+eIF4A	16 ± 4	0.995	17 ± 2	0.997	>7000
eIF4GI_557-1599_+eIF4A + ATP	14 ± 2	0.973	13 ± 3	0.963	7000
eIF4GI_557-1599_+eIF4A + eIF4E	15 ± 6	0.991	14 ± 4	0.996	520 ± 15
eIF4GI_557-1599_+eIF4A + eIF4E + eIF4B	14 ± 4	0.963	19 ± 6	0.967	
eIF4GI_557-1599_+eIF4A + eIF4E + ATP	18 ± 3	0.966	22 ± 1	0.983	136 ± 11
eIF4GI_557-1599_+eIF4A + eIF4E + ADP	13 ± 4	0.954	26 ± 3	0.960	1018 ± 22
eIF4GI_557-1599_+eIF4A + eIF4B	24 ± 5	0.991	12 ± 3	0.973	
eIF4GI_557-1599_+eIF4A + eIF4B + ATP	19 ± 3	0.987	17 ± 3	0.993	
eIF4GI_557-1599_+eIF4A + eIF44B + AMPPNP	20 ± 5	0.985	14 ± 3	0.991	
eIF4GI_682-1599_	120 ± 7	0.998	139 ± 23	0.998	
eIF4GI_682-1599_+eIF4E	110 ± 25	0.998	142 ± 17	0.992	
eIF4GI_682-1599_+eIF4A	26 ± 3	0.982	17 ± 5	0.955	1056 ± 101
eIF4GI_682-1599_+eIF4A + ATP	16 ± 4	0.977	20 ± 3	0.968	321 ± 71
eIF4GI_682-1599_+eIF4A + eIF4E	20 ± 2	0.962	21 ± 5	0.993	
eIF4GI_682-1599_+eIF4A + eIF4E + ATP	30 ± 4	0.980	16 ± 5	0.983	

χ^2^ represents the goodness of fit.

a Data adapted from Izidoro *et al.* 2022.

### eIF4A, but not eIF4E enhance the binding of eIF4GI_682-1599_ to uncapped 5′UTRs

For comparison, binding to a truncated form of eIF4GI, eIF4GI_682-1599_ (amino acids 682–1599) that lacks the autoinhibitory domain eIF4E was measured. As expected, adding exogenous eIF4E did not affect the binding interaction of eIF4GI_682-1599_ (denoted as eIF4GI_682-1599_•4E complex) to the uncapped 5′UTRs of FGF-9 (K_D_, 110 ± 25 nM) (Table 1) and HIF-1α (K_D_, 142 ± 17 nM) (Table 1) mRNAs, compared to eIF4GI_682-1599_ alone (K_D_, 120 ± 7 nM and 139 ± 23 nM for uncapped 5′UTRs of FGF-9 and HIF-1α mRNAs, respectively) (Table 1). Interestingly, eIF4A enhanced the binding affinity of eIF4GI_682-1599_ to the uncapped 5′UTR mRNAs. The eIF4GI_682-1599_•4A complex bound to the uncapped 5′UTR of FGF-9 mRNA with a K_D_ of 26 ± 3 nM, a 4.6-fold increase in binding affinity compared with eIF4GI_682-1599_ alone (K_D_, 120 ± 7 nM) (Table 1). Similarly, the eIF4GI_682-1599_•4A complex bound to the 5′UTR of HIF-1α mRNA with a K_D_ of 17 ± 5 nM, an almost 8-fold increase in binding affinity compared to eIF4GI_682-1599_ alone (Table 1). These results suggest that eIF4A acts synergistically with eIF4GI_682-1599_ to form a higher-affinity binding complex with the uncapped 5′UTRs of FGF-9 and HIF-1α mRNAs. The binding affinities of eIF4GI_682-1599_, eIF4A, and eIF4E complex (denoted as eIF4GI_682-1599_•4A•4E) with the uncapped 5′UTR of FGF-9 (K_D_, 20 ± 2 nM) and HIF-1α (K_D_, 21 ± 5 nM) mRNAs were determined. The eIF4E did not show any additional enhancement of eIF4GI_682-1599_•4A•4E complex binding to uncapped 5′UTR mRNAs, compared to eIF4GI_682-1599_•4A complex. Further, while eIF4A enhanced binding affinity, it did not increase selectivity between the two 5′UTRs.

### ATP hydrolysis is not required for recruitment of eIF4GI to the uncapped 5′UTRs of FGF-9 and HIF-1α encoding mRNAs

**Figure 2:**
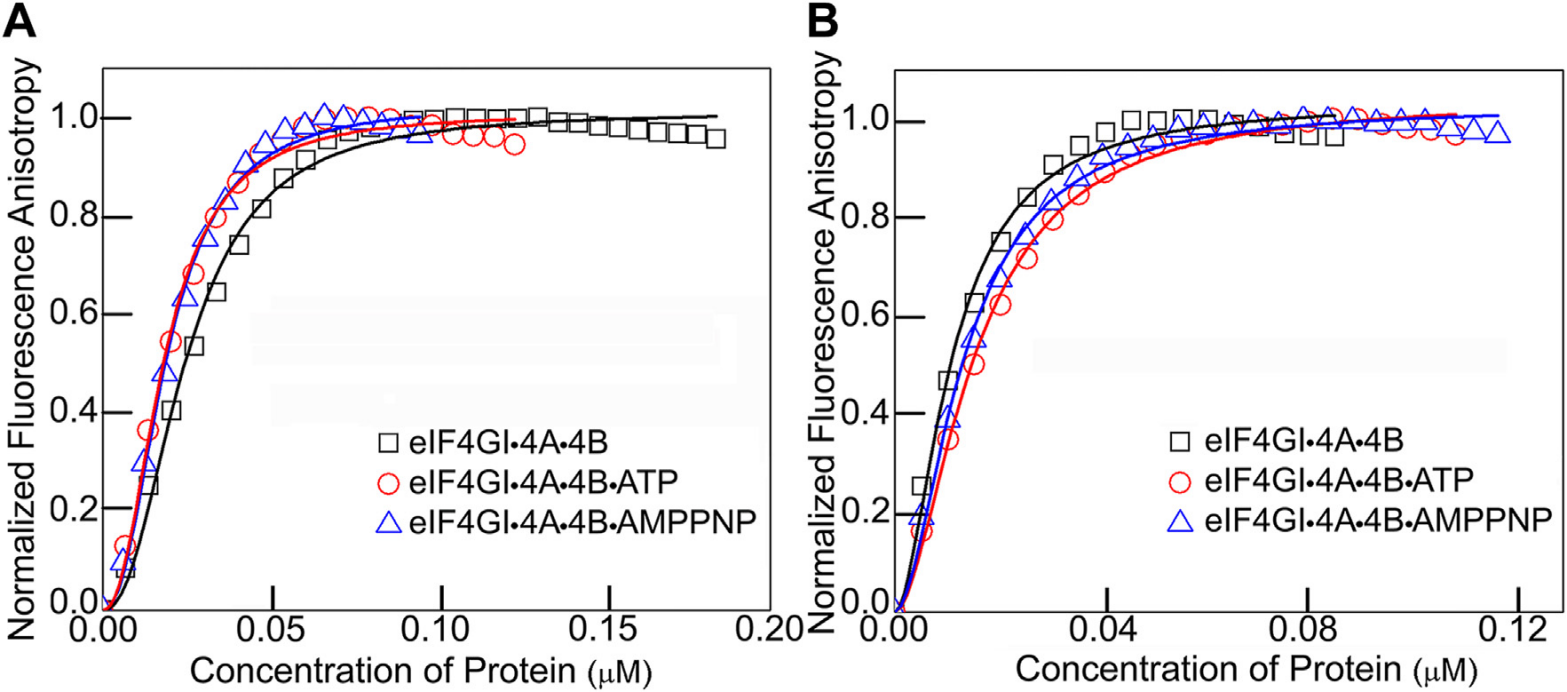
**Equilibrium binding titrations of uncapped 5′UTR mRNAs to the helicase complex.** Normalized anisotropy changes of 10 nM fluorescein-labeled uncapped 5′UTRs of (*A*) FGF-9 and (*B*) HIF-1α mRNAs binding to eIF4GI_557-1599_•eIF4A•eIF4B in the absence of nucleotide, or the presence of ATP or nonhydrolyzable AMP-PNP. Data points correspond to the average of three independent anisotropy measurements. The curves represent the non-linear fits used to obtain the corresponding K_D_ values. eIF, eukaryotic initiation factor FGF-9, fibroblast-growth factor 9; HIF-1α, hypoxia inducible factor 1 subunit alpha.

To examine the effect of ATP hydrolysis and eIF4A helicase activity on the recruitment of eIF4GI to these mRNAs, fluorescein labeled uncapped 5′UTRs of FGF-9 and HIF-1α mRNAs were preincubated with eIF4A, eIF4B, and ATP for 30 min and then titrated with increasing amounts of eIF4GI_557-1599_. Fluorescence based anisotropy results show that ATP hydrolysis is not required to improve the binding affinity of eIF4GI_557-1599_ to either uncapped 5′UTRs of FGF-9 or HIF-1α mRNAs (Table 1, Fig. 2, *A* and *B*). The helicase complex without ATP (eIF4GI_557-1599_•4A•4B) had a 4-fold increased binding affinity to the uncapped 5′UTR of HIF-1α mRNA (K_D_, 12 ± 3 nM) compared to eIF4GI_557-1599_ alone (K_D_, 50 ± 6 nM) (Table 1, Fig. 2*B*). In contrast, eIF4GI_557-1599_•4A•4B binding affinity to the uncapped 5′UTR of FGF-9 mRNA (K_D,_ 24 ± 5 nM) was 2-fold weaker than that obtained for eIF4GI_557-1599_ alone (K_D_, 14 ± 3 nM) (Table 1, Fig. 2*A*). Further, to determine if ATP hydrolysis and helicase activity were important, AMP-PNP, a nonhydrolyzable ATP analog was used to determine binding affinities of uncapped 5′UTRs of FGF-9 (eIF4GI_557-1599_•4A•4 B•AMPPNP, K_D_, 20 ± 5 nM) and HIF-1α (eIF4GI_557-1599_•4A•4B•AMPPNP, K_D_, 14 ± 3 nM) mRNAs, respectively (Table 1, Fig. 2, *A* and *B*). These surprising results suggest that eIF4A enhance recruitment of eIF4GI to the uncapped 5′UTRs of FGF-9 and HIF-1α mRNAs, but ATP hydrolysis is not required.

### The nucleotide bound state of eIF4A does not enhance 5′UTR mRNA recruitment of eIF4GI

**Figure 3:**
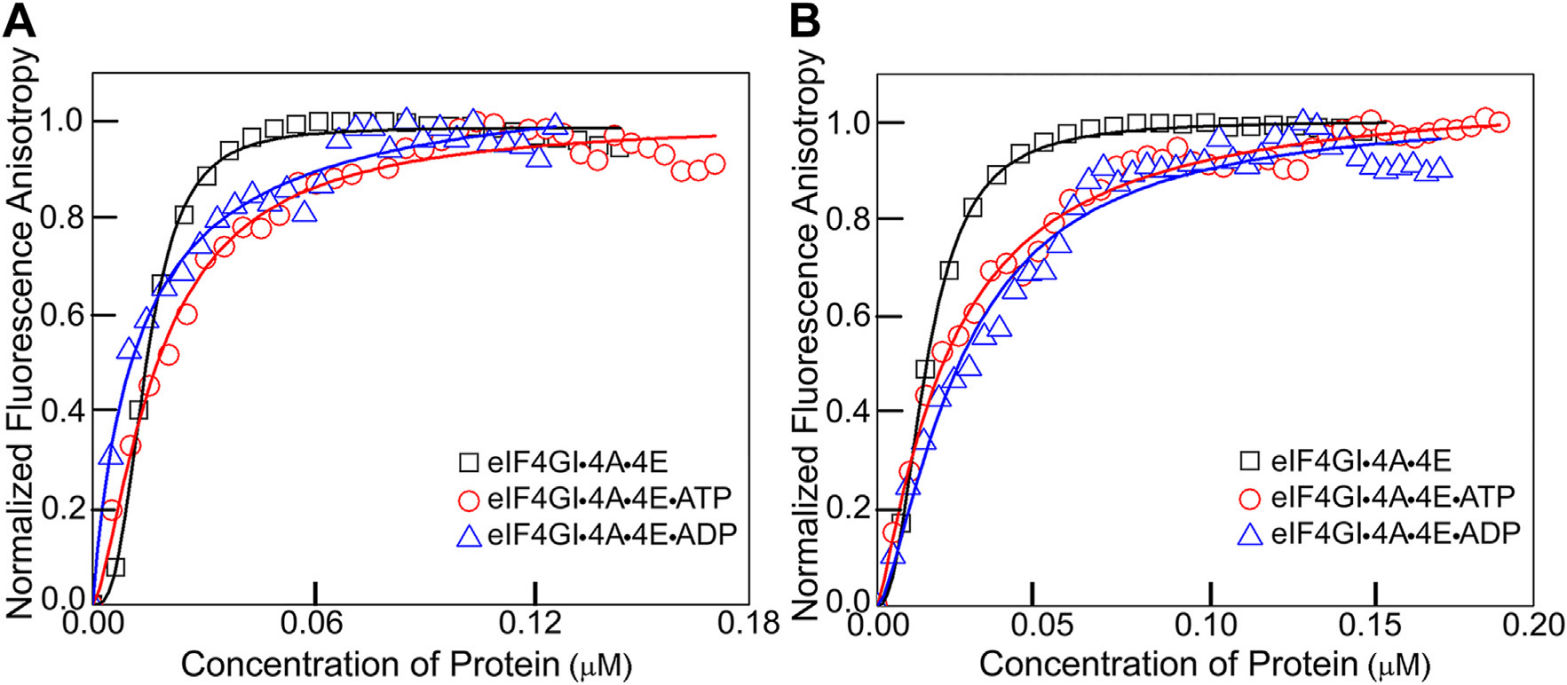
**Effect of ATP and ADP on binding of eIF4G-4A-4E complexes to FGF-9 and HIF-1α 5′UTR-mRNAs.** Equilibrium binding titrations of fluorescein-labeled uncapped 5′UTRs of (*A*) FGF-9 and (*B*) HIF-1α mRNAs binding to eIF4GI_557-1599_•eIF4A•eIF4E complex in the absence of nucleotide, or the presence of saturating amount of ATP or ADP. Average of three independent experiments was performed and the curves represent the nonlinear fits that were used to obtain the averages and standard deviations for the corresponding K_D_ values. eIF, eukaryotic initiation factor; FGF-9, fibroblast-growth factor 9; HIF-1α, hypoxia inducible factor 1 subunit alpha.

The binding affinity of human eIF4A to single-stranded unstructured RNA is 40-fold greater when bound to ATP compared to ADP (41, 42). Therefore, to determine if the nucleotide-bound state of eIF4A also regulates the binding of highly structured, uncapped 5′UTR mRNAs with eIF4F, the binding affinities of each uncapped 5′UTR mRNA to eIF4GI_557-1599_•4A•4E in the presence of ATP, ADP, and a nonhydrolyzable analog were measured. Fluorescence assays were performed by titrating eIF4GI_557-1599_ into a fixed amount of each fluorescently labeled uncapped 5′UTR mRNA (10 nM) in the presence of 1 μM eIF4A, 1 μM eIF4E, and 1 mM ATP. The dissociation constants for the binding of the uncapped 5′UTR of FGF-9 mRNA to eIF4GI_557-1599_•4A•4E•ATP complex was 18 ± 3 nM, compared to 13 ± 4 nM for the ADP complex (eIF4GI_557-1599_•4A•4 E•ADP), respectively (Table 1, Fig. 3*A*). Similarly, for uncapped 5′UTR of HIF-1α mRNA the K_D_ values were 22 ± 1 nM and 26 ± 3 nM for the ATP and ADP complexes, respectively (Table 1, Fig. 3*B*). Further, the binding affinities of eIF4GI_557-1599_•4A•4E without nucleotide to the uncapped 5′UTR of FGF-9 (K_D,_ 15 ± 6 nM) and HIF-1α (K_D,_ 14 ± 4 nM) mRNAs (Table 1, Fig. 3, *A* and *B*) were determined. ATP-bound eIF4A had little effect on the binding of uncapped 5′UTR of FGF-9 mRNA to eIF4F. On the other hand, a 2-fold reduction was observed when the uncapped 5′UTR of HIF-1α mRNA interacts with eIF4GI_557-1599_•4A•4E in the presence of ATP. In our experimental setup a 10 nM fluorescence probe (labeled mRNA) was used. K_D_’s below 10 nM cannot be reliably determined. Thus, the reason for these small changes in the binding affinities may not completely reflect the changes in the affinity since binding is already very tight. Our anisotropy assay is also limited by the eIF4GI•eIF4A•eIF4E concentrations. We only consider the eIF4GI•eIF4A•eIF4E concentrations in the range that do not cause any abnormal anisotropy changes due to aggregation or precipitation. We also determined the binding affinity of uncapped 5′UTRs of FGF-9 and HIF-1α mRNAs to eIF4A in the presence of ATP to understand the degree of ATP-dependent RNA binding. Surprisingly, uncapped 5′UTRs of FGF9 and HIF-1α mRNAs showed only 5.8-fold and 9-fold greater binding affinities to eIF4A when bound to nucleotide, respectively (Table 1). The eIF4A itself had little effect on increasing the binding affinity of these uncapped 5′UTR mRNAs to eIF4GI_557-1599_. These results contrast with the effects previously reported for an uncapped, unstructured 42nt RNA (37).

The binding affinities were also measured using a truncated form of human eIF4GI, eIF4GI_682-1599,_ which lacks the eIF4E binding domain and mimics a poliovirus 2A protease cleaved form of the protein (36). The equilibrium dissociation constants for eIF4GI_682-1599_•4A•ATP binding to the uncapped 5′UTRs of FGF-9 (K_D,_ 16 ± 4 nM) and HIF-1α (K_D,_ 20  ± 3 nM) mRNAs were similar to those in the absence of ATP (K_D,_ 26  ± 3 and 17 ± 5 nM for 5′UTR of FGF-9 and HIF-1α mRNAs, respectively) (Table 1). ATP had a minimal effect on the binding of eIF4GI_682-1599_ to uncapped mRNAs. Taken together, all these data indicate a mechanism where the nucleotide-bound state of eIF4A does not regulate eIF to the highly structured uncapped RNA tested here.

### The uncapped 5′UTRs of FGF-9 and HIF-1α encoding mRNAs induce different structural changes in the eIF4GI-eIF4A-eIF4E-RNA complexes

**Figure 4:**
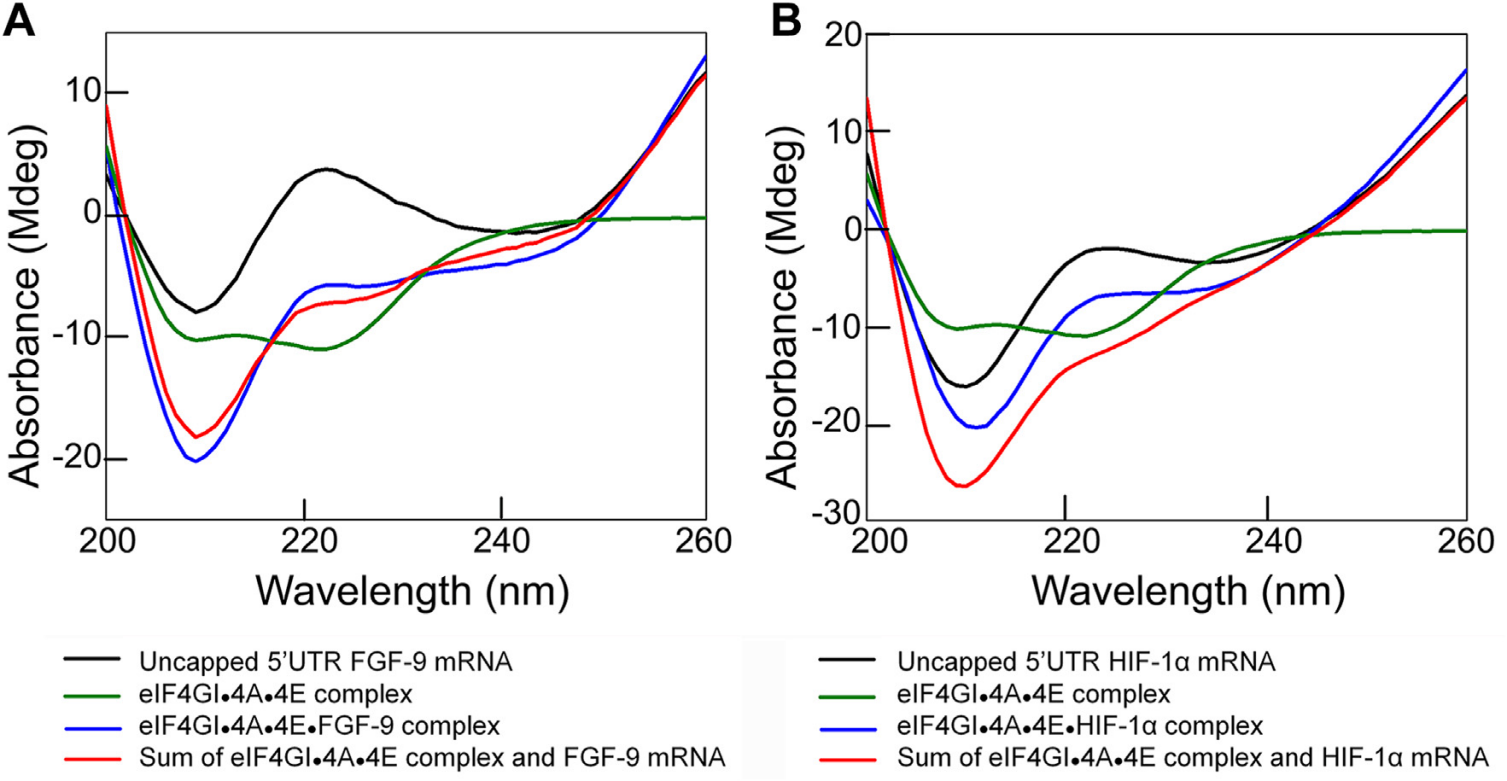
**CD spectral comparison of eIF interactions with FGF-9 and HIF-1α encoding mRNAs.** The CD spectra of uncapped 5′UTRs of (*A*) FGF-9 and (*B*) HIF-1α encoding mRNAs and their interaction with eIF4GI_557-1599_•eIF4A•eIF4E protein complex, and the sum of eIF4GI_557-1599_•eIF4A•eIF4E complex spectra and 5′UTR mRNA spectra are as shown. FGF-9, fibroblast-growth factor 9; HIF-1α, hypoxia inducible factor 1 subunit alpha; eIF, eukaryotic initiation factor.

To further probe the interaction between the uncapped 5′UTR mRNAs and multiprotein eIFs complexes, CD spectroscopy was performed and the conformational change resulting from the interactions between the uncapped 5′UTR mRNAs and proteins were measured (Fig. 4, *A* and *B*). For CD spectra (24),

$$\Delta A=A_{R}-A_{L}=\left(\varepsilon_{R}-\varepsilon_{L}\right)cl=\Delta \varepsilon cl$$

where A is absorbance, ε is the molar extinction coefficient, the subscripts R and L refer to right and left circular polarized light, respectively, *c* is the molar concentration, and *l* is the path length. For a mixture, the observed absorbance is,

$$\Delta A=\Delta \varepsilon cl_{1}+\Delta \varepsilon cl_{2}$$

where subscripts 1 and 2 refer to the two different components. At a given wavelength, the sum of the ΔA for individual protein and RNA will be equal to the ΔA for the mixture if no interaction has occurred. Our previous report showed that eIF4GI_557-1599_ induces conformational changes in eIF4GI_557-1599_•mRNA complex while interacting with FGF-9 and HIF-1α mRNAs (28). Interestingly, binding of uncapped 5′UTR of FGF-9 and HIF-1α mRNAs to eIF4A•eIF4E complex also leads to notable conformational changes. Further interaction with eIF4GI_557-1599_ to eIF4A•eIF4E complex along with HIF-1α possess a significant structural change in eIF4GI_557-1599_•4A•4E•HIF-1α complex. Fig. 4*B* showed a reduced signal for the eIF4GI_557-1599_•eIF4A•eIF4E•uncapped 5′UTR HIF-1α complex (denoted as eIF4GI_557-1599_•4A•4E•HIF-1α) compared to the sum of the eIF4GI_557-1599_•4A•4E complex and the uncapped 5′UTR of HIF-1α mRNA. Therefore, the binding of the uncapped 5′UTR of HIF-1α mRNA induces secondary structural changes in the eIF4GI_557-1599_•4A•4E•RNA complex (reminiscent of the mammalian eIF4F complex) (29), consistent with the improved binding affinity of the uncapped 5′UTR of HIF-1α mRNA to this complex compared to eIF4GI alone in the binding assays (Fig. 1*B*). In contrast, binding of uncapped 5′UTR of FGF-9 mRNA to eIF4A•eIF4E complex induces conformational changes in such a way that further interactions with eIF4GI_557-1599_ to eIF4A•eIF4E along with FGF-9 mRNA (eIF4GI_557-1599_•4A•4E•FGF-9) minimize the overall conformational changes compared to eIF4GI_557-1599_ alone. Thus, it suggests that eIF4A•eIF4E interaction cancel out the overall conformational changes of eIF4GI_557-1599_•4A•4E•FGF-9 complex. As a result, the binding of the uncapped 5′UTR of FGF-9 mRNA to the eIF4GI_557-1599_•4A•4E protein complex showed a smaller effect, with only slight spectra changes (Fig. 4*A*), which is consistent with its binding affinity (Fig. 1*A*).

### eIF4A stimulates cap-independent translation of FGF-9 and HIF-1α ApppG-capped-UTR-Luc-mRNAs

**Figure 5:**
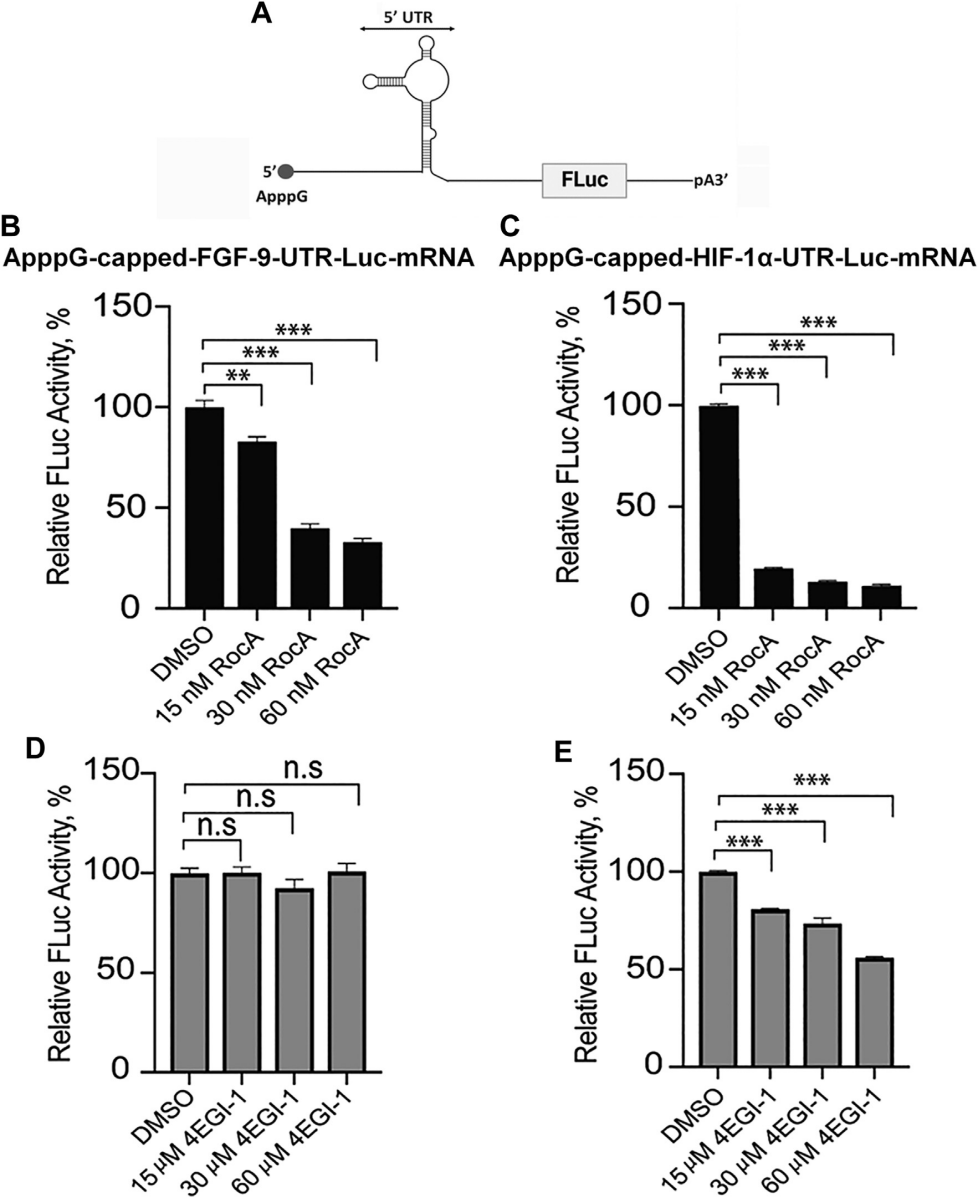
**RocA and 4EGI-1 differentially affect the cap-independent activities of FGF-9 and HIF-1α ApppG-capped-UTR-Luc-mRNA reporters.***A*, cartoon representation of the mRNA reporter used for this study. Translation yields of (*B*) ApppG-capped-FGF-9-UTR-Luc-mRNA and (*C*) ApppG-capped-HIF-1α-UTR-Luc-mRNA were monitored in nuclease-treated RRL supplemented with DMSO (control) or increasing concentrations of RocA. Translation yields of (*D*) ApppG-capped-FGF-9-UTR-Luc-mRNA and (*E***)** ApppG-capped-HIF-1α-UTR-Luc-mRNA were monitored in nuclease-treated RRL supplemented with DMSO (control) or increasing concentrations of 4EGI-1. Bar heights and error bars correspond to the average and standard deviations, respectively, of three independent luciferase activity measurements with control (DMSO) set at 100%. Data were analyzed by two-tailed unpaired Student’s *t* test: n.s, *p* < 0.033; ∗∗, *p* = 0.002; ∗∗∗, *p* < 0.001. FGF-9, fibroblast-growth factor 9; HIF-1α, hypoxia inducible factor 1 subunit alpha; RRL, rabbit reticulocyte lysate; RocA, Rocaglamide; DMSO, dimethylsulfoxide.

To gain further insight into the functional role of eIF4A in the cap-independent translation for FGF-9 and HIF-1α encoding mRNAs, *in vitro* translation assays using nuclease-treated rabbit reticulocyte lysate (RRL) were performed. The effects of an eIF4A inhibitor, Rocaglamide (RocA) were tested on their translation efficiencies. RocA inhibits the unwinding activity of eIF4A by increasing its affinity for the RNA in a homopurine-dependent manner, thereby impeding the scanning complexes (43, 44). Though RocA has been suggested to make eIF4A function as a roadblock on A-rich sequences and may therefore not specifically inhibit eIF4A in the initiation complex. Also, it has been shown that secondary structure in 5′ untranslated regions is only a minor determinant for RocA selectivity and that RocA does not repress translation by reducing eIF4A availability. Rather, *in vitro* and in cells, RocA specifically clamps eIF4A onto polypurine sequences in an ATP-independent manner. This artificially clamped eIF4A further blocks 43S scanning, leading to premature, upstream translation initiation, and reducing protein expression. Thus, RocA inhibits the translation initiation in 5′UTR mRNAs by blocking the 43S scanning in an eIF4A dependent manner. Luciferase reporter constructs were generated for each 5′UTR (UTR-Luc-mRNA, Fig. 5*A*) and capped with a nonfunctional cap-analog (ApppG). The expression levels of following addition of RocA (Fig. 5, *B* and *C*) were measured. Analysis of the relative firefly luciferase expression for each ApppG-capped-UTR-Luc-mRNA candidate, showed a noticeable decrease in translation activity with increasing concentrations of RocA. A much higher concentration of RocA was required to achieve the same degree of inhibition for ApppG-capped-FGF-9-UTR-Luc-mRNA (Fig. 5*B*) compared to ApppG-capped-HIF-1α-UTR-Luc-mRNA (Fig. 5*C*). Specifically, at a RocA concentration of 60 nM, a 80 to 90% decrease in translation yield was observed for ApppG-capped-HIF-1α-UTR-Luc-mRNA, while only 17 to 67% reduction in translation activity was observed for ApppG-capped-FGF-9-UTR-Luc-mRNA with the addition of 60 nM RocA.

**Figure 6:**
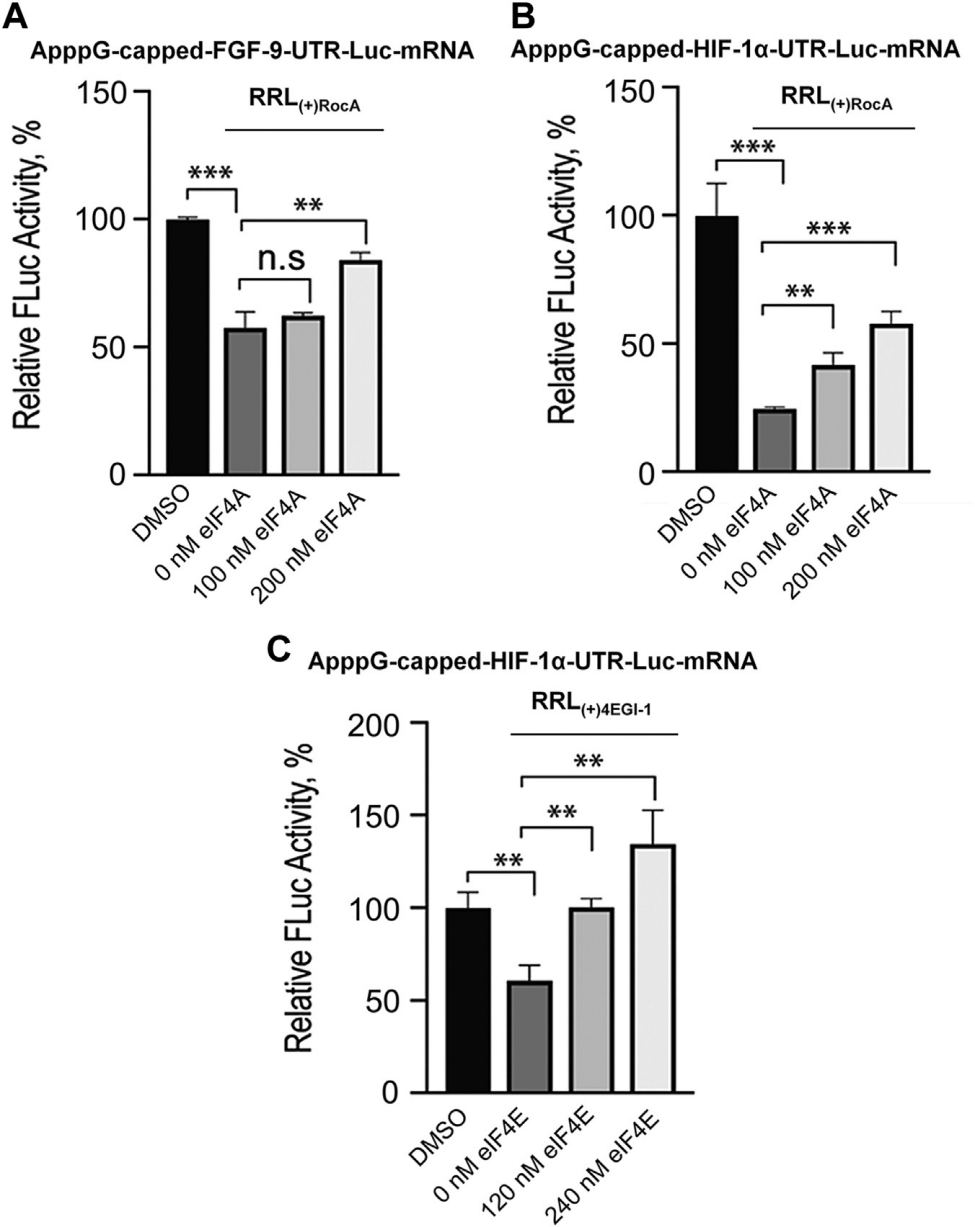
**Effect of eIF4A and eIF4E on the cap-independent activities of FGF-9 and HIF-1α ApppG-capped-UTR-Luc-mRNA reporters.** Translation yields of (*A*) ApppG-capped-FGF-9-UTR-Luc-mRNA and (*B*) ApppG-capped- HIF-1α-UTR-Luc-mRNA following treatment of the RRL with 60 nM of Rocaglamide (RRL_(+)RocA_) and increasing concentration of eIF4A. *C*, translation yields of ApppG-capped-HIF-1α-UTR-Luc-mRNA following treatment of the RRL with 60 μM 4EGI-1 molecule (RRL_(+)4EGI-1_) and increasing concentration of eIF4E. Bar heights and error bars correspond to the average and standard deviations, respectively, of three independent luciferase activity measurements with control (DMSO) set at 100%. Data were analyzed by two-tailed unpaired Student’s *t* test: n.s, *p* = 0.12; ∗, *p* < 0.033; ∗∗, *p* = 0.002; ∗∗∗, *p* < 0.001. FGF-9, fibroblast-growth factor 9; HIF-1α, hypoxia inducible factor 1 subunit alpha; eIF, eukaryotic initiation factor; RRL, rabbit reticulocyte lysate; DMSO, dimethylsulfoxide.

To further investigate whether the decrease in translation yield of FGF-9 and HIF-1α ApppG-capped-UTR-Luc-mRNA reporters by RocA was specific and due to eIF4A inhibition, we tested if the addition of purified exogenous eIF4A could rescue cap-independent translation activities. When eIF4A was inhibited by 60 nM RocA, an increase in the cap-independent translation efficiencies of both FGF-9 and HIF-1α ApppG-capped-UTR-Luc-mRNAs was observed following addition of purified eIF4A (Fig. 6, *A* and *B*). These results indicate that eIF4A was required for the optimal cap-independent translation of these mRNAs. Importantly, it has been speculated that the helicase activity of eIF4A is required for the translation of all mRNAs regardless of their structural complexity (45).

As a control, the effect of RocA inhibitor on the control reporter construct m^7^G-capped-β-actin-UTR-Luc-mRNA was examined. As expected, RocA significantly reduced the translation activity of m^7^G-capped-β-actin-UTR-Luc-mRNA up to 83% (Fig. S1*A*). To test specificity, exogeneous purified eIF4A was added to the 60 nM RocA-treated m^7^G-capped-β-actin-UTR-Luc-mRNA in RRL lysate (RRL_(+)RocA_) which showed a recovery of 67 to 72% translation activity (Fig. S1*A*).

### eIF4E differentially affects cap-independent translation of FGF-9 and HIF-1α ApppG-capped-UTR-Luc-mRNAs

To investigate the functional role of eIF4E in the cap-independent translation of both FGF-9 and HIF-1α encoding mRNAs, *in vitro* translation assays were performed in the presence of eIF4E inhibitor, 4EGI-1. 4EGI-1 is a small molecule that has been identified to bind eIF4E and disrupt eIF4E/eIF4GI association (46). In response to addition of 4EGI-1 to RRL lysate (RRL_(+)4EGI-1_), the translation efficiency of ApppG-capped-FGF-9-UTR-Luc-mRNA was not significantly impacted (Fig. 5*D*). In contrast, the addition of 4EGI-1 resulted in significant reduction in translation levels of ApppG-capped-HIF-1α-UTR-Luc-mRNA, reducing it to 20 to 45% of the control without the inhibitor (Fig. 5*E*). The functional role of eIF4E in the cap-independent translation of ApppG-capped-HIF-1α-UTR-Luc-mRNA reporter was investigated, given that the 4EGI-1 molecule inhibited its translation but not that of ApppG-capped-FGF-9-UTR-Luc-mRNA (Fig. 5, *D* and *E*). Following treatment of the lysate with 4EGI-1 (RRL_(+)4EGI-1_), the translation of ApppG-capped-HIF-1α-UTR-Luc-mRNA was restored by addition of exogenous eIF4E (Fig. 6*C*) indicating the specificity of the inhibitor and the effect of eIF4E.

The effect of 4EGI-1 inhibitor on the control reporter construct m^7^G-capped-β-actin-UTR-Luc-mRNA was also examined. Translation activity of m^7^G-capped-β-actin-UTR-Luc-mRNA was reduced to 88% by 4EGI-1 inhibitor. While addition of exogenous purified eIF4E to the 4EGI-1-treated m^7^G-capped-β-actin-UTR-Luc-mRNA in RRL lysate (RRL_(+)4EGI-1_) resulted in 79 to 86% stimulation of translation activity (Fig. S1*B*).

## Discussion

In this study, we have used fluorescence-based steady state anisotropy assays, CD studies and *in vitro* translation assays to explore how different eukaryotic initiation factors (eIF4GI, eIF4A, eIF4E, and eIF4B) are recruited to the highly structured 5′UTRs of cellular mRNAs to drive cap-independent translation initiation. Our previous reports showed that, during cap-independent translation, eIF4GI recognizes and binds to the specific structural elements within the 5′ UTRs of FGF-9 and HIF-1α mRNAs, stimulating cap-independent translation initiation (27). We also showed that a free 5′-end of ApppG-capped-HIF-1α-UTR-Luc-mRNA was necessary for scanning the 43S PIC to initiate translation, suggesting a CITE-like cap-independent translation initiation mechanism (16, 27). Here, we identified eIF4A and eIF4E as additional key factors, along with eIF4GI, that differentially drive cap-independent translation of a subset of human mRNAs. We found that eIF4GI_557-1599_•4A and eIF4GI_557-1599_•4E complexes both bound to the uncapped 5′UTR of HIF-1α mRNA with 3-fold greater binding affinities than eIF4GI_557-1599_ alone (Fig. 1*B*). This increase in eIF4GI binding to RNA upon addition of eIF4E is consistent with previous findings in plant and virus IRES. Specifically, the binding of eIF4E to an autoinhibitory domain of plant eIF4G increases the binding affinity of eIF4G for a 3′cap-independent translation element of BYDV (BTE) by 2.5-fold to 6-fold, depending on the different eIF4G truncations used (24), suggesting that the eIF4E binding domain is important in regulating RNA binding to eIF4F. Moreover, the eIF4E binding domain enhances the binding affinity of eIF4GI to a poliovirus IRES mutant that lacks domain V (denoted as PV dV) (36). The K_D_ value calculated from a fluorescence anisotropy assay was 276 ± 21 nM for eIF4GI_557-1599_ bound to PV dV, while the K_D_ value was reduced to 49 ± 2 nM with the addition of eIF4E, indicating a positive cooperative binding effect between eIF4GI and eIF4E on the PV dV (36). This cooperative binding effect is likely due to eIF4E-induced conformational changes in eIF4GI, which enhance its RNA-binding affinity and specificity. Additionally, eIF4E promotes mRNA restructuring of a highly structured uncapped duplex RNA by stimulating eIF4A helicase activity, which plays a significant role in cap-independent translation initiation (35).

The multiprotein complex eIF4GI_557-1599_•4A•4E had 3.5-fold greater binding affinity to the uncapped 5′UTR of HIF-1α mRNA compared to eIF4GI_557-1599_ alone. Our CD data confirmed the significant conformational changes in the eIF4GI_557-1599_•4A•4E complex in the presence of uncapped 5′UTR of HIF-1α mRNA (Fig. 4*B*). Previous report indicated that the presence of the eIF4E-binding domain in eIF4GI_557-1599_ induced conformational rearrangements in the eIF4GI_557-1599_ and/or RNA (29), thereby increasing the binding affinity of eIF4GI to 5′UTR mRNAs (28). Other reports have similarly shown high binding affinity of eIF4G in the presence of eIF4A for encephalomyocarditis virus IRES (47). Furthermore, eIF4E binding to the eIF4G has been demonstrated to increase the binding affinity of eIF4G for BYDV BTE mRNA (24). Interestingly, neither eIF4A nor eIF4E improved the binding affinity of eIF4GI_557-1599_ for uncapped 5′UTR of FGF-9 mRNA, suggesting that eIF4GI_557-1599_ alone is sufficient for stable binding (Fig. 1*A*). Consistent with the binding data, our CD data showed no conformational changes in the eIF4GI_557-1599_•4A•4E complex with the uncapped 5′UTR of FGF-9 mRNA (Fig. 4*A*), further supporting that eIF4GI_557-1599_ alone can bind stably without additional factors. This suggests that the 5′UTR of FGF-9 mRNA acts as an IRES-like translational enhancer rather than a CITE-like translational enhancer, possibly requiring fewer eIFs for ribosome recruitment to its start codon (27).

It has been generally accepted that the helicase activity of eIF4A plays a critical role in unwinding mRNA secondary structure to generate a single-stranded region that can be accommodated into the 40S subunit decoding site (48). A recent report from the Fraser group demonstrated that approximately 50% of the duplex unwinding activity on a reporter mRNA occurred in an eIF4A-independent manner (48). Our observation clearly demonstrated that eIF4A might be necessary for unwinding the secondary structure of the 5′UTRs of FGF-9 and HIF-1α mRNAs, but not ATP hydrolysis (Fig. 2, *A* and *B*). The Fraser group also showed that ATP hydrolysis did not have any role in recruiting a 42nt unstructured mRNA to the 43S PIC (45, 49). This suggests that ATP hydrolysis by eIF4A is independent of its requirement for translation of many mRNAs, regardless of the degree of structure in their 5′UTR (45, 50, 51). In contrast, for globin-Luc-mRNA, ATP hydrolysis was stringently needed, as it is thought that ATP was required by eIF4A for unwinding the secondary structure of globin reporter at the 5′UTR (45). Our results indicate that eIF4A plays a dual role in translation, enhancing assembly of the initiation complex even when translation does not directly involve cap binding, and stimulating the helicase activity by unwinding the mRNA secondary structures.

The role of nucleotide-bound state of eIF4A in the cap-independent translation of the 5′UTRs of FGF-9 and HIF-1α mRNAs was further elucidated. Previous studies have shown that ATP binding and hydrolysis induces a cycle of conformational rearrangement and changes in RNA affinity for eIF4A (41, 42, 52). These conformational changes enable eIF4A to bind and release RNA. The affinity of human eIF4A for single-stranded RNA is 40-fold greater when the protein is bound to ATP compared to ADP (41). For unstructured mRNAs, the nucleotide-bound state of eIF4A significantly dominates the association and dissociation rate constants of RNA binding to eIF4F (37). It was observed that the binding affinity for 42nt unstructured RNA to eIF4GI_557-1599_•4A•4E was 7-fold greater in the presence of ATP compared to ADP (37). Here, we show that structured 5′UTR mRNAs behave very differently. Specifically, we observed a 2-fold reduction in binding affinity when uncapped 5′UTR of HIF-1α mRNA binds to eIF4GI_557-1599_•4A•4E in the presence of ATP. In contrast, no effect of the nucleotide was observed on the binding of structured 5′UTR of FGF-9 mRNA to eIF4GI_557-1599_•4A•4E (K_D_ = 15 ± 6 nM) in the presence of ATP (K_D_ = 18 ± 3 nM), and ADP (K_D_ = 13 ± 4 nM), (Fig. 3, *A* and *B*). Similar trends were found with a truncated form of eIF4GI, eIF4GI_682-1599,_ that lacks the eIF4E binding domain. There was no effect of ATP on the binding of structured 5′UTR of FGF-9 or HIF-1α mRNAs compared to in the absence of ATP (Table 1). In contrast, a 3-fold reduction was observed in the equilibrium dissociation constant for unstructured RNA CAA-42-FL binding to the eIF4GI_682-1599_•4A complex compared with CAA-42-FL binding to the eIF4GI_682-1599_•4A•ATP complex (37). Therefore, our results indicate that secondary structure in 5′UTRs of FGF-9 and HIF-1α mRNAs interacts differently with eIF4A compared to unstructured mRNAs. Interestingly, these highly structured 5′UTR mRNAs do not require eIF4A to be in its ATP-bound state for recruiting eIF4F. It appears that the structural elements within these 5′UTR may circumvent the need for eIF4A•ATP, at least in terms of binding affinity.

The functional roles of eIF4A in the cap-independent activities of FGF-9 and HIF-1α ApppG-capped-UTR-Luc-mRNAs were further supported by *in vitro* translation assays where addition of eIF4A inhibitor, Rocaglamide, inhibited the translation output of these mRNA transcripts (Fig. 5, *B* and *C*). Interestingly, similar concentrations of RocA had varied inhibitory effects on the cap-independent translation of FGF-9 and HIF-1α ApppG-capped-UTR-Luc-mRNA reporters (Fig. 5, *B* and *C*). This suggests that eIF4A has complex roles in the recruitment of the 43S PIC. eIF4A plays multiple roles in the protein initiation pathway (45), including unwinding mRNA secondary structures within the vicinity of the start codon (30) and helicase-independent recruitment of mRNA to the ribosome (45). Thus, inhibiting its activities in the lysate disrupted the cap-independent translation of both 5′UTRs of FGF-9 and HIF-1α mRNAs. The eIF4A inhibitor, RocA, reduced translation of ApppG-capped-HIF-1α-UTR-Luc-mRNA more effectively than for ApppG-capped-FGF-9-UTR-Luc-mRNA, consistent with our binding studies and demonstrates the requirement for eIF4A for cap-independent translation of both mRNAs. These results, when combined with the binding and ATP-dependent data, suggest that HIF-1α mRNA requires some degree of scanning, potentially in an ATP-independent manner as some helicase activity can occur without ATP hydrolysis (48). Intriguingly, although eIF4A did not improve the binding affinity of eIF4GI_557-1599_ to the uncapped 5′UTR of FGF-9 mRNA, its cap-independent activities were inhibited in the lysate treated with RocA (Fig. 5*B*). This suggests that other roles of eIF4A in translation such as the helicase-independent recruitment of the PIC and modulating the conformation of the 40S ribosomal subunit may have been affected by RocA (45). Therefore, we speculate that eIF4A’s role in IRES-like cap-independent translation may involve assisting in loading of the 40S subunit onto the uncapped 5′UTR of mRNA. These results support the roles of eIF4A in translation of all mRNAs (30, 31). Our results also identified eIF4E as an important factor for the cap-independent translation of ApppG-capped-HIF-1α-UTR-Luc-mRNA but not ApppG-capped-FGF-9-UTR-Luc-mRNA. Consistent with previous data which showed that eIF4GI_557-1599_, which contains the eIF4E binding domain, restored the translation activity of eIF4GI-depleted RRL lysates for the ApppG-capped-UTR-Luc-mRNA transcript (27). While, a truncated eIF4GI_682-1599_ which lacks the eIF4E binding domain was less effective in restoring translation, indicating that stimulation of translation was due to the direct interaction of eIF4GI with IRES/CITE elements in the 5′UTRs of mRNAs. Although it is established that cap-independent translation is eIF4E-independent (16), it is likely that the binding of eIF4E to eIF4GI may cause a conformational change in eIF4GI complex that enhance its binding to the uncapped 5′UTR of HIF-1α mRNA as well as stimulates translation activity. This is supported by our fluorescence binding assays (Table 1, Fig. 1*B*), CD spectra (Fig. 4*B*), and *in vitro* translation assays (Figs. 5 and 6). Additionally, eIF4E has also been shown to stimulate eIF4A helicase activity and promote mRNA restructuring (35), which may promote a CITE-like mechanism of uncapped 5′UTR of HIF-1α but is not essential for an IRES-like mechanism of uncapped 5′UTR of FGF-9.

**Figure 7:**
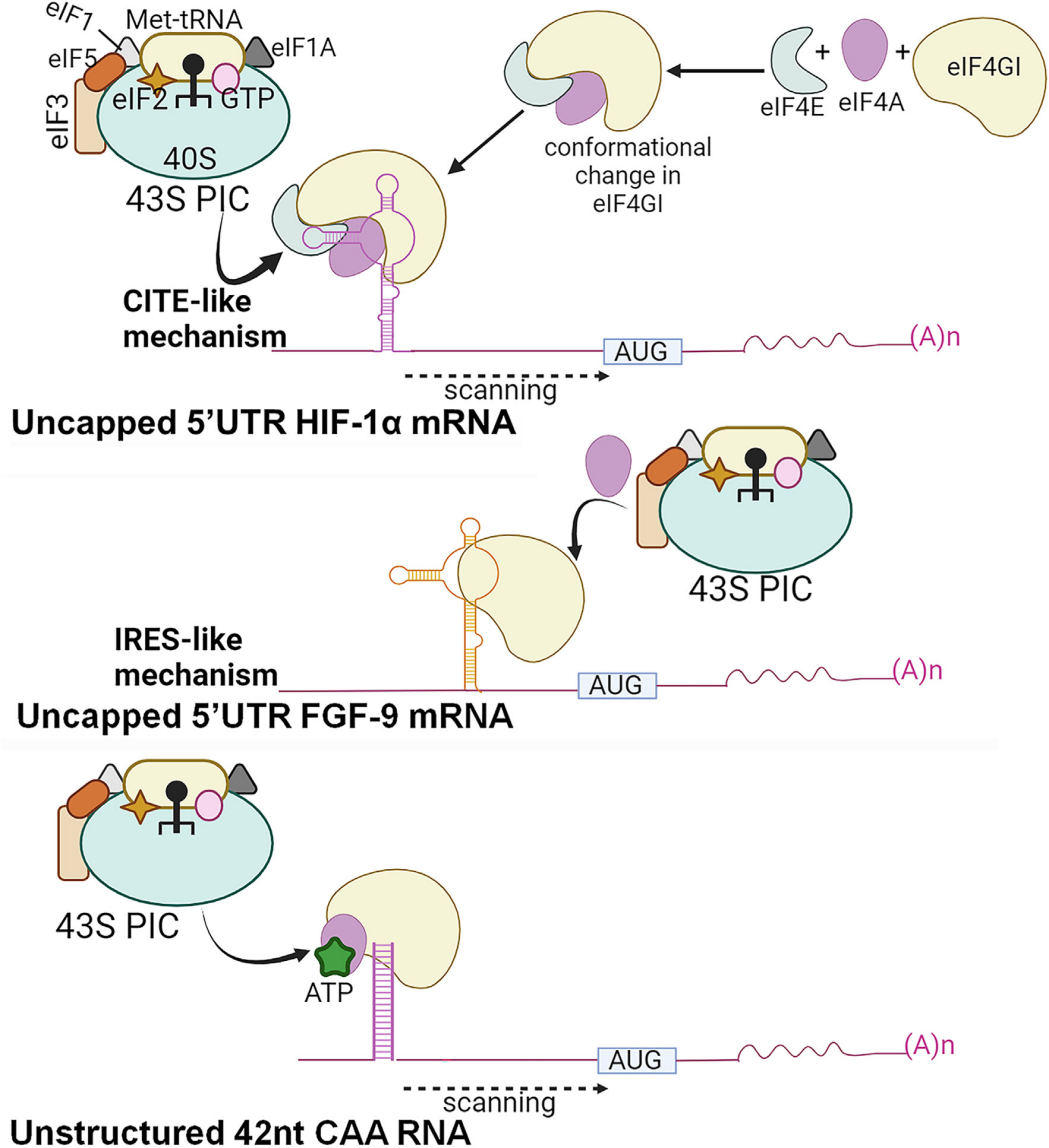
**Proposed model describing two possible mechanisms of cap-independent translation initiation of 5′UTRs of HIF-1α and FGF-9 encoding mRNAs employed by cells to mitigate cellular stress conditions.** Under stress, cap-dependent translation is compromised due to m^7^G cap unavailability caused by the sequestration of eIF4E by hypophosphorylated 4E-binding proteins (4E-BP1) (8, 27). *Top* panel shows when bound by eIF4A and eIF4E, eIF4GI protein undergoes a conformational change that enhances binding to CITE*-*elements in the HIF-1α 5′UTR. Using the CITE-like pathway, HIF-1α 5′UTR recruits the 43S PIC at or near its 5′ end. This complex scans downstream until it encounters a start codon and begins translation (*Top* panel). In contrast, FGF-9 mRNA uses its IRES-like element to stably bind eIF4GI, which allows it to recruit the 43 S PIC within the vicinity of its start codon and initiate translation with little or no scanning (*Middle* panel). *Bottom* panel showed nucleotide-bound eIF4A regulates the recruitment of unstructured RNA to eIF4GI. The 43S PIC scans through 5′ to 3′ on mRNA until it recognizes a start codon, facilitating translation initiation (Modified from Izidoro *et al.* 2022). CITE, cap-independent translation enhancer; IRES, internal ribosome entry site; eIF, eukaryotic initiation factor; FGF-9, fibroblast-growth factor 9; HIF-1α, hypoxia inducible factor 1 subunit alpha; m^7^G, methyl guanosine; PIC, preinitiation complex.

In summary, this work highlights the presence of multiple cap-independent mechanisms involving different initiation factor requirements used by cellular mRNAs. Further, these eIFs play multiple roles depending on which mRNAs are being expressed. Given all the above, we propose a more detailed description of two possible cap-independent translation mechanisms used by cells to mitigate cellular stress conditions (Fig. 7). When bound by eIF4A and eIF4E, eIF4GI undergoes a conformational change that enhances its binding to CITE*-*elements in the 5′UTR of HIF-1α mRNA. Using the CITE-like pathway, the 5′UTR of HIF-1α mRNA recruits the 43S PIC at or near its 5′ end and scans downstream until it encounters a start codon and initiates translation (Fig. 7, Top panel). In contrast, the 5′UTR of FGF-9 mRNA uses its IRES-like element to stably bind eIF4GI, which enables it to recruit the 43S PIC within the proximity of its start codon and initiate translation. Therefore, the IRES-like mechanism, which involves little or no scanning, requires fewer eIFs to initiate protein synthesis (Fig. 7, Middle panel). This model provides an attractive mechanism through which IRES- or CITE-mRNAs bypass stress-induced shutdown of cap-dependent translation, undergoing eIF4GI-mediated cap-independent initiation and expression aided by different factors. Moreover, unstructured mRNAs follow different translation initiation mechanisms, where the nucleotide bound state of eIF4A regulates the recruitment of mRNA to eIF4F (37). The eIF4F complex further binds to 43S PIC and translation initiates *via* 5′ to 3′ scanning on mRNA until a start codon is recognized (Fig. 7, Bottom panel). These multiple roles for eIFs depending on RNA structure highlight the complexity of protein synthesis regulation.

## Experimental procedures

### Purification of recombinant eIF4GI, eIF4A, eIF4B, and eIF4E

Human eIF4GI_557-1599_, eIF4GI_682-1599_, and eIF4E clones were a generous gift from Dr Christopher S. Fraser. The human eIF4B clone was obtained from Dr Ruben Gonzalez Jr’s lab (Columbia University). eIF4GI_557-1599_ and eIF4GI_682-1599_ were recombinantly expressed and purified as previously described (27). The eIF4A clone contained an N-terminal His-tag and maltose binding protein-tag (MBPfusion constructs) followed by a tobacco etch virus (TEV) protease cleavage site. The eIF4B clone contained only an N-terminal His-tag. Briefly, both eIF4A and eIF4B clones were recombinantly expressed from BL21 (DE3) cells. Induction of proteins was carried out using 0.5 mM IPTG for 3 h at 30 °C after the absorbance (*A*) reached an absorbance value of 0.5 at 595 nm. Next, recombinant His-tagged eIF4A and eIF4B were then purified from the bacterial cell lysates using His-trap HP (Ni-NTA) columns according to the manufacturer’s instructions (53). The purified proteins with 6x histidine tags were dialyzed overnight at 4 °C against the storage buffer (20 mM Hepesz pH 7.3, 200 mM KCl, 1 mM DTT, and 10% glycerol) to remove excess imidazole. Concurrently, TEV protease was added to cleave and separate the histidine tags from the proteins. Following the overnight digestion with TEV protease at 4 °C to cleave off the tags, the proteins without tags were further purified. This purification was achieved by passing the proteins through 1 ml HiTrap Heparin HP columns (Cytiva), which efficiently separated the cleaved histidine tags and maltose binding protein-tag from the eIF4A protein. Post column purification, the resulting proteins were subjected to analysis using 10% SDS-PAGE gels. Fractions exhibiting a purity greater than 95% as judged by SDS-PAGE as discribed below, were combined and subsequently dialyzed overnight against storage buffer for storage at −80 °C. The human eIF4E/pCDF-duet clone was designed for the coexpression of two target genes. The eIF4E was cloned in the second ORF of this vector. It had an N-terminal polyhistidine tag, followed by a TEV protease cleavage site to allow for subsequent removal of the tag from eIF4E protein. For expression of recombinant eIF4E protein, *Escherichia coli* Rosetta (DE3) cells were transformed with this clone and grown in 1.5 L of LB medium. The bacterial cells were grown at 37 °C until the *A*_600_ reached 0.6 to 0.8. Protein expression was induced overnight at 20 °C, by adding 0.5 mM IPTG. The cells were pelleted and sonicated in the lysis buffer (25 mM Hepes pH 7.5, 300 mM KCl, 10% glycerol, 1 mM DTT, and 20 mM imidazole, with added protease inhibitor tablet). The supernatant was filtered through a 400-micron syringe filter and then applied to a 5 ml Ni-NTA column that had been preequilibrated with the same lysis buffer. The column was washed with 20 ml buffer E (25 mM Hepes pH 7.5, 300 mM KCl, 10% glycerol, 1 mM DTT, and 50 mM imidazole), and the protein was eluted with buffer E with an increased imidazole concentration of 500 mM. Fractions containing eIF4E were pooled and dialyzed overnight at 4 °C against buffer E (without any imidazole) containing TEV protease to cleave the His-tag from eIF4E. After dialysis, the now untagged protein was further purified using a 5 ml Q-Sepharose column to remove the cleaved tag and the TEV protease. Briefly, the KCl concentration of dialyzed samples was adjusted to 100 mM, and the sample was loaded on a 5 ml preequilibrated Q-Sepharose column (preequilibrated with buffer A- 25 mM Hepes pH 7.5, 100 mM KCl, 10% glycerol, and 1 mM DTT). A step gradient elution with 150 to 500 mM KCl in buffer A was used to purify the proteins. Fractions containing untagged eIF4E, were collected, pooled, concentrated, and stored at −80 °C. Aliquots of purified proteins were analyzed using 10% SDS-PAGE gel and quantified using Bradford’s assay. Concentrated proteins (Fig. S2) were aliquoted and stored at −80 °C in the storage buffer.

### *In vitro* RNA transcription and 3′-fluorescein labeling

DNA templates of 5′UTR of FGF-9 (177 nts, GenBank accession number: AY682094.1) (27, 28, 54) and 5′UTR of HIF-1α (294 nts, GenBank accession number: AH006957.2) (27, 28, 55) were purchased from Integrated DNA Technology. All the DNA oligonucleotides were transcribed using the T7 transcription kit (New England Biolabs Inc) following the manufacturer’s protocol. Next, transcribed RNAs were purified with Zymo Research Clean and Concentration Kit following the manufacture’s protocol. The purities of the RNAs were verified by 1.5% gel electrophoresis and the sample concentrations were determined by nano-drop UV-visible spectrometer.

The RNAs were labeled at their 3′ end using sodium acetate and sodium peroxide at pH 5.2, and incubated for 30 min in the dark (56) with the reaction mixture covered with aluminum foil. Then, 1M sodium sulfite was added to the reaction mixture, and incubated for another 10 min in the dark. The oxidized RNA was purified by ethanol precipitation. For labeling, 1 mM flouroscein-5-thiosemicarbazide and 50 mM Na-phosphate buffer (pH 6.5) were added to the oxidized RNA followed by incubating the reaction mixture for 2 h in the dark. Next, 1 M NaCNBH_3_ was added and the reaction mixture was incubated overnight at 4 ^o^C. Finally, the labeled RNA was purified using a two-step purification method that involved ethanol precipitation and RNA Clean and Concentrator Kit by following the manufacturer’s protocol. RNA concentrations were measured using a Nano-drop UV/visible spectrometer, and their integrity was verified by 1.5% agarose gel electrophoresis.

### Fluorescence anisotropy binding assays

Fluorescence-based anisotropy assays were carried out to assess the binding of the 5′UTR mRNAs to different eukaryotic initiation factors (eIF4GI, eIF4A, eIF4E, and eIF4B) both in the presence and absence of nucleotide. Equilibrium anisotropy assays were performed using a stopped-flow (model SF-300X, KinTek Corporation) set up equipped with a titration module and a temperature controller (Thermo Fisher Scientific), as previously described (19, 27). Fluorescein-labeled RNAs were excited at 495 nm wavelength and emission was detected using a 515 nm high-pass filter (Semrock). Briefly, anisotropy binding titrations were carried out with a 200 μl sample containing 10 nM fluorescein-labeled mRNA in the titration buffer (20 mM Hepes–KOH, 200 mM KCl, pH 7.6, and 1 mM MgCl_2_). Subsequently, 20 μl of 2.5 μM protein or protein complexes were injected into the sample at 25 ± 0.5 °C, as described in the results section. The final concentration of the other components was also saturated according to the available affinities and the titration range (1 μM eIF4A, 1 μM eIF4E, and 1 mM ATP) used. Saturation was verified by titrating each fluorescence labeled mRNA (at a fixed concentration) with different concentrations of protein/protein complex without observing a change in binding affinity. Equilibrium is also verified by running each titration at different time duration without noticing change in the K_D_ values. Fifty data points were collected for each titration over a period of 30 min, where the first reading was taken in the absence of protein. Prior to titration, all labeled mRNAs were heated to 90 ^o^C for 2 min, slowly cooled to room temperature over 1 h, followed by addition of MgCl_2_ (1 mM final concentration). The solutions were then incubated on ice for 1 h. The equilibrium dissociation constants (K_D_’s) were calculated by fitting the Hill equation using Origin Pro8 software (https://www.originlab.com/demodownload.aspx),

$$r_{\text{obs}}=r_{\min}+\left(r_{\max}-r_{\min}\right)\left[\frac{{\left[P\text{rotein}\right]}^{n}}{{K_{D}}^{n}+{\left[\text{Protein}\right]}^{n}}\right]$$

where r_obs_ is the observed anisotropy value, r_min_ is the minimum anisotropy value in the absence of protein (eIF4GI, eIF4A, or eIF4E), r_max_ is the final saturated anisotropy value, [Protein] is the concentration of eIF4GI, eIF4A, or eIF4E, K_D_ is the equilibrium dissociation constant and n is the number of binding sites. Here n is equal to 1 ± 0.15. The chi-squared values (χ^2^) that represented the statistical goodness of fit were always close to 1 (Table 1). Fitting data to a two-site model (n = 2) did not improve the fit as judged by (χ^2^) values. The equilibrium binding titration of each 5′UTR was performed three times and fitted independently for K_D_. The fitted K_D_^’^s were then averaged, and the standard deviations were calculated (Table1).

### CD spectral study

All CD measurements were carried out using an AVIV CD spectrometer (AVIV Biomedical, Model 202–01) equipped with a Peltier controller system (thermal controller Thermo Neslab Merlin M33 connected to a water bath). Spectra were recorded at 25 ± 0.5 ^o^C over the wavelength range of 200 to 260 nm with a 1 nm bandwidth, using 0.1 cm optical path length quartz cuvettes. An average of five scans was taken for each spectrum. Ellipticity measurements were corrected against a buffer baseline using the same cuvette. For each CD measurement, 1 μM protein with a saturated mRNA concentration in a 10 mM phosphate buffer was used. eIF4GI_557-1599_, eIF4E, and eIF4A were preincubated to form the protein complex before scanning. The helical content of the protein complexes was measured in terms of mean-residue ellipticity (deg cm^2^ dmol^-1^) as reported (24).

MRE (θ) = observed CD (mdeg) / (*cnl* x 10)

where *c* represents the protein concentration in molarity, *n* is the number of amino-acid residues, and *l* denotes the cuvette path length.

### Preparation of UTR-Luc reporter mRNAs

The UTR-Luc mRNA constructs for the luciferase gene expression reporter assays were generated from the BlucB plasmid according to reported protocols (27, 57). The BlucB plasmid contains a firefly luciferase gene flanked by 5′- and 3′ UTR sequences of the BYDV genomic RNA. Each UTR-Luc mRNA reporter construct includes a T7 promoter sequence followed by the target 5′ UTR, which was cloned into the BlucB plasmid vector upstream of the firefly luciferase coding region, after removing the BYDV 5′UTR as reported previously (27). DNA templates were transcribed *in vitro* using T7 RiboMax Large Scale RNA Production Kit (Promega) following the manufacturer’s protocol. ApppG Cap Analog (NEB) or Ribo m^7^GpppA Cap Analog (Promega) were added to the transcription mix in an ApppG:GTP or Ribo m7GpppA:GTP ratio of 10:1 to obtain mRNA transcripts with nonfunctional and functional caps, respectively. Capped RNAs were poly(A) tailed (pA) using the Poly(A) Tailing Kit (Invitrogen) following the manufacturer’s protocol. The resulting capped and polyadenylated mRNAs were then purified using the Monarch RNA Cleanup Kit following the manufacturer’s protocol. RNA concentrations were measured using nano-drop UV/visible spectrometer, and the integrity was verified by 1.0% agarose gel electrophoresis.

### Luciferase-based gene expression reporter assays

Gene expression was carried out by performing *in vitro* translation assay of the UTR-Luc mRNAs, using the nuclease treated RRL (Promega). Each 25 μl translation reaction mixture contained 70% v/v of RRL (Promega) supplemented with 0.5 mM MgCl_2_, 0.02 mM amino acid mixture, 10 U/μl RiboLock RNase Inhibitor (Thermo Fisher Scientific), and varying concentrations of purified eIF4A and eIF4E proteins in presence of RocA (2 mM stock in dimethylsulfoxide (MedChemExpress, catalog number HY-19356) and 4EGI-1 chemical (10 mM stock in dimethylsulfoxide) (Selleck Chemicals, catalog number S7369), (Figs. 5 and 6). Briefly, 1 μg (60 nM) of 5′UTR-Luc-mRNA construct was added to the RRL *in vitro* translation mixture following the addition of the specific concentration of RocA and eIF4A or 4EGI-1 and eIF4E. The resulting *in vitro* translation reaction mixture was then incubated at 30 °C for 1 h, and the reaction was stopped by placing on ice. Firefly luciferase activities were then measured with SpectraMax iD5 Multi-Mode Microplate reader (Molecular Devices). Luminescence was measured in the illuminometer by adding 5 μl of the translation reaction to 40 μl Bright-Glo Luciferase assay reagent (Promega), and reading the output in the luminometer with a spectral wavelength range of 350 to 650 nm and an integration time of 10s at room temperature. After subtracting the background, the luminescence data were analyzed and plotted using the GraphPad Prism 9 software package (https://www.graphpad.com/). Three different batches of RRLs were used, and translation data for each UTR-Luc-mRNA in different RRL batches was normalized with respect to m^7^G-capped-β-actin-UTR-Luc-mRNA transcript. For our experimental conditions, the luciferase activity for FGF-9 and HIF-1α ApppG-capped-UTR-Luc-mRNA varied from 3000 to 7000 a.u. But for m^7^G-capped-β-actin-UTR-Luc-mRNA, the luciferase activity ranges from 50,000 to 60000 a.u. for different RRL batches. Each UTR-Luc-mRNA translation datum was reported as an average of three independent experiments. Each independent experiment was done in triplicate and the mean ± SD was calculated using GraphPad Prism 9. Statistical significance between the mean values was analyzed using two-tailed unpaired student’s *t* test (GraphPad Prism 9 software). *p*-values were calculated using a statistical significance threshold of 0.05.

## Data availability

All data are present in the manuscript and its supporting information file.

## Supporting information

This article contains supporting information.

## Conflict of interest

The authors declare that they have no conflicts of interest with the contents of this article.
