# Supplementary material for: Mechanistic differences in eukaryotic initiation factor requirements for eIF4GI-driven cap-independent translation of structured mRNAs
Source: J Biol Chem. 2024 Oct 9;300(11):107866. doi: 10.1016/j.jbc.2024.107866 (PMC11570956; doi:10.1016/j.jbc.2024.107866)
Supplement: Supporting Information [file mmc1.docx]

**Supporting Information**

**Mechanistic differences in eukaryotic initiation factor requirements for eIF4GI-driven cap-independent translation of structured mRNAs**

Baishakhi Saha^1^, Solomon A. Haizel^2,3^ and Dixie J. Goss^1,2,4,^*

***^1^*** *Department of Chemistry, Hunter College, City University of New York, New York, NY 10065*

*^2^ Ph.D. Program in Biochemistry, The Graduate Center of the City University of New York, New York, NY 10016*

*^3^Center for Genomics and Systems Biology, New York University, New York, NY 10003*

*^4^Ph.D. Program in Chemistry, The Graduate Center of the City University of New York, New York, NY 10016*

* Corresponding author:

 Dixie J. Goss

Email: dgoss@hunter.cuny.edu

**Items included:**

**Figure S1.** *In vitro* translation assay.

**Figure S2.** SDS-PAGE gel.

**Figure S1**.





**Figure S1.** **Inhibition of m^7^G-capped-β-actin-UTR-Luc-mRNA by Rocaglamide or 4EGI-1.** **(A)** Bar plots represent the translation yields of m^7^G-capped-β-actin-UTR-Luc-mRNA following treatment of the RRL with 60 nM Rocaglamide (RRL_(+)RocA_) and increasing concentration of eIF4A as indicated. **(B)** Bar plots represent the translation yields of m^7^G-capped-β-actin-UTR-Luc-mRNA following treatment of the RRL with 60 nM 4EGI-1 (RRL_(+)4EGI-1_) and increasing concentration of eIF4E as indicated. Bar heights and error bars correspond to the average and standard deviations, respectively, of three independent luciferase activity measurements with control (DMSO) set at 100%. Data were analyzed by two-tailed unpaired Student’s *t*-test: **, p =0.002; ***, p < 0.001; ****, p < 0.0001.

**Figure S2.**

**
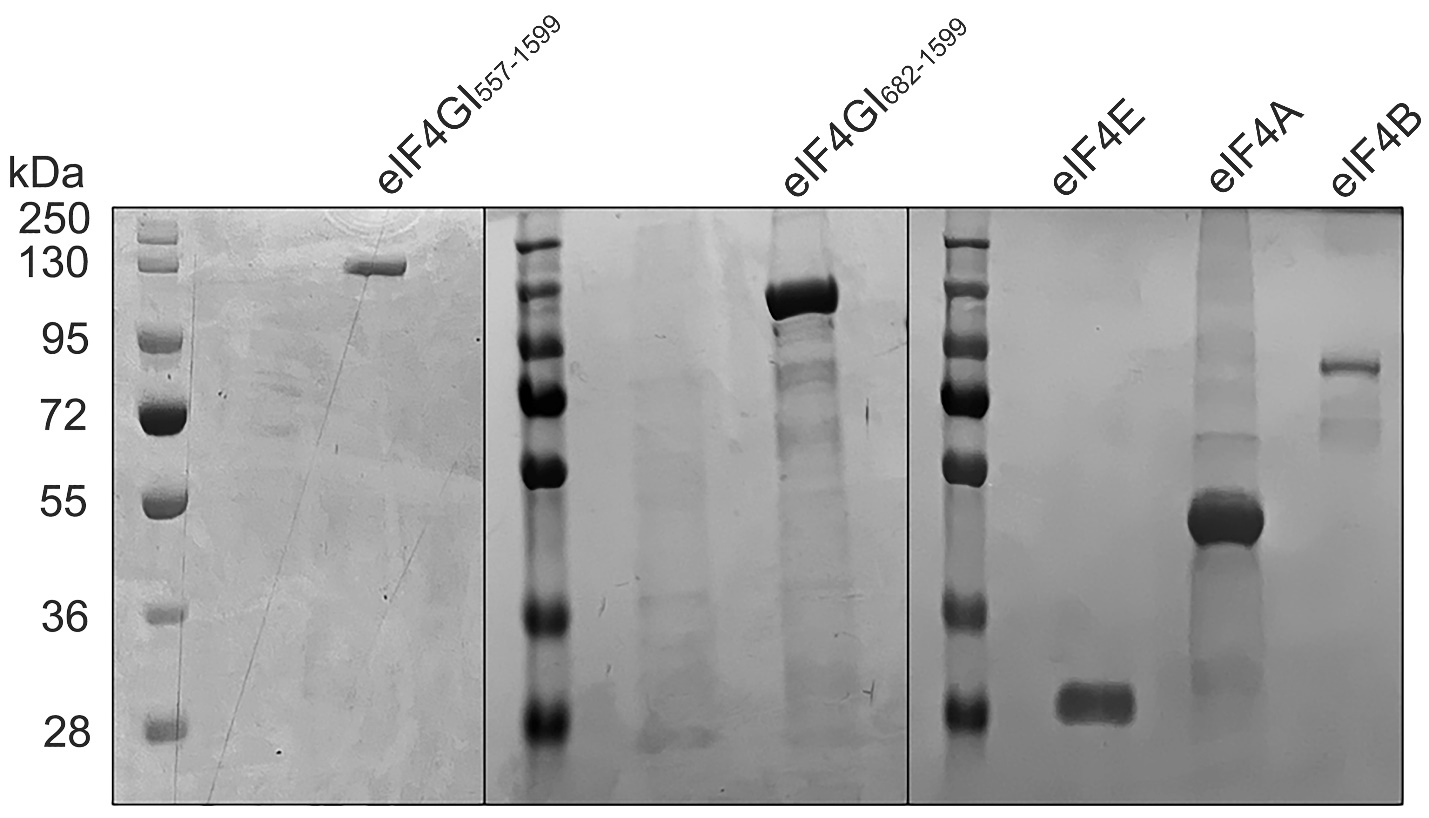
**

**Figure S2**. A 10% SDS-PAGE gel showing purity of eIF4GI_557-1599_, eIF4GI_682-1599,_ eIF4E, eIF4A and eIF4B, used for this study.
